# Emulsion-Templated Oleogels from Citrus Fiber and Pumpkin Seed Oil By-Product as Palm Oil Substitutes in Chocolate Sauce

**DOI:** 10.3390/foods15132272

**Published:** 2026-06-25

**Authors:** Sumeyra Cimen, Zeynep Hazal Tekin-Cakmak, Salih Karasu, Hatice Bekiroglu, Mustafa Tahsin Yilmaz, Osman Sagdic

**Affiliations:** 1 Department of Food Engineering, Faculty of Chemical and Metallurgical Engineering, Yildiz Technical University, 34210 Istanbul, Türkiye; 2 Department of Food Engineering, Faculty of Agriculture, Sirnak University, 73300 Sirnak, Türkiye; 3 Department of Industrial Engineering, Faculty of Engineering, King Abdulaziz University, Jeddah 21589, Saudi Arabia

**Keywords:** emulsion-templated oleogel, citrus fiber, texture profile analysis, fat-structuring strategy, oxidative stability, fat substitute

## Abstract

Oleogel-based fat systems were developed using citrus fiber (CF) and cold-pressed pumpkin seed oil by-product (PSB) through an emulsion-template approach and evaluated as palm oil substitutes in a model chocolate sauce system. Oleogels were prepared by varying CF (4–5%) and PSB (0–2%) concentrations and characterized in terms of rheological, textural, and sensory properties and oxidative stability. The emulsions exhibited predominant elastic behavior (G′ > G″), with storage modulus (K′) values increasing from 694.12 to 2242.54 Pas^n^ as CF and PSB concentrations increased. Chocolate sauces formulated with CF–PSB oleogels showed pseudoplastic flow behavior and solid-like viscoelastic characteristics, with K′ values ranging from 89.32 to 356.56 Pas^n^, compared to 34.84 and 16.95 Pas^n^ for the palm oil (C1) and sunflower oil (C2) controls, respectively. Temperature sweep and thermal loop tests demonstrated improved thermal resistance and emulsion stability in oleogel-containing chocolate sauces. Oleogel-based chocolate sauces also showed superior emulsion stability under thermal cycling and greater oxidative stability compared to the C2 sample. Texture profile analysis revealed increased hardness and spreadability with higher CF and PSB contents, consistent with rheological findings. Oleogel-based chocolate sauces also demonstrated enhanced oxidative stability, with induction period values reaching 18:55 h compared to 5:13 h for the sunflower oil control and approaching the palm oil control (20:13 h). Texture profile analysis revealed increased hardness and spreadability with higher CF and PSB contents. Sensory evaluation indicated that sauces containing 5% CF and 1–2% PSB received the highest scores for flavor, consistency, and overall acceptability.

## 1. Introduction

Trans and saturated fats are widely used in processed foods because of their desirable technological properties. However, their excessive consumption has been associated with cardiovascular diseases, obesity, type 2 diabetes, and metabolic syndrome [1,2,3]. Consequently, developing healthier fat systems without compromising product quality remains a major challenge in food formulation. Although liquid vegetable oils offer nutritional advantages, their direct use often results in poor structural integrity and undesirable textural properties due to the lack of a solid fat network [4]. To address this limitation, oleogels have emerged as promising fat-structuring systems that impart solid-like characteristics to liquid oils [5].

Oleogels can be produced using direct or indirect (emulsion-templated) methods [6]. While the direct method involves dispersing oleogelators such as beeswax, monoacylglycerols, or ethyl cellulose directly into oil at elevated temperatures, this approach is constrained by the limited availability of food-grade oleogelators and the increased risk of lipid oxidation [6,7]. The emulsion-templated approach enables the incorporation of hydrophilic biopolymers through an intermediate aqueous phase, thus providing greater formulation flexibility and expanding the range of biopolymers suitable for oleogel production [8].

Citrus fiber (CF) is obtained from citrus peel through organic solvent-assisted extraction followed by drying. CF is primarily composed of cellulose, hemicellulose, pectin, and protein networks. This complex biopolymer structure confers high water- and oil-holding capacities as well as viscosity-enhancing properties; therefore, CF is a suitable structuring agent for emulsion-based systems [9,10,11]. Pumpkin seed oil by-product (PSB) obtained from pumpkin seed oil extraction contains high levels of protein, fiber, and bioactive compounds, which supports its valorization as a functional and sustainable ingredient for food applications [12]. Together, these characteristics make CF and PSB promising components for the development of emulsion-templated oleogels. Polysaccharide-rich agro-industrial by-products have attracted increasing attention as functional ingredients for fat replacement due to their ability to improve water-binding capacity, oil retention, emulsion stability, and network formation in complex food matrices. Their fibrous and polymeric structures can enhance viscosity, strengthen dispersed systems, and support the formation of gel-like networks, thereby contributing to the technological functionality required in reduced-fat or structured fat food formulations. Although emulsion-templated oleogels have shown potential as alternative fat systems, their application in complex food matrices remains limited. In particular, the use of CF and PSB together as sustainable structuring materials for chocolate sauce formulations has not been adequately investigated.

Chocolate sauces are widely consumed with various food products such as fruit salads, ice cream, and desserts. Palm oil, which is frequently used in the formulation of these sauces, is preferred due to its neutral taste, functional properties and solid structure. Palm oil is commonly used in the food industry because of its high melting point and its ability to remain solid at room temperature, making it one of the few plant-based oils with these properties. However, its high saturated fat content (approximately 50%) is a concern from a nutritional perspective [13,14]. Therefore, developing palm oil alternatives that provide comparable technological functionality while offering improved nutritional and sustainability profiles is of considerable interest.

The purpose of this study was to develop CF-PSB-based emulsion-templated oleogels and evaluate their feasibility as palm oil substitutes in a model chocolate sauce system. The effects of CF–PSB emulsion-templated oleogels on the physical, structural, and sensory properties of sauce formulation were investigated. The present study demonstrates a process-oriented and sustainable fat-structuring strategy for a model chocolate sauce system.

## 2. Materials and Methods

### 2.1. Materials

Cocoa powder, sunflower oil, and sugar were purchased from a local market in Türkiye, while palm oil was obtained from a local supplier. Citrus fiber was supplied by Plentia Superfoods (Istanbul, Türkiye). Pumpkin seed oil by-product (PSB), defined as the solid fraction remaining after cold-pressed pumpkin seed oil extraction, was provided by ONEVA Foods Company (Istanbul, Türkiye). Xanthan gum was procured from Sigma-Aldrich (Sigma Chemical Co., St. Louis, MO, USA).

### 2.2. Methods

#### 2.2.1. Preparation of Oleogels

Oleogels were prepared using an emulsion-template process with minor modifications from the method reported by Qiu, C.; Huang, Y.; Li, A.; Ma, D.; Wang, Y. [15]. Citrus fiber (CF, 4–5%, *w*/*w*) and pumpkin seed oil by-product (PSB, 0–2%, *w*/*w*) were dispersed in water at 60 °C to form hydrogels. The hydrogels (55%, *w*/*w*) were then homogenized with sunflower oil (45%, *w*/*w*) using a high-shear homogenizer at 12,000 rpm for 3 min to obtain oil-in-water emulsions. The emulsions were frozen at −80 °C and subsequently freeze-dried to remove the aqueous phase.

#### 2.2.2. Preparation of Chocolate Sauces

Chocolate sauces were formulated based on preliminary trials. Powdered sugar was ground and sieved, and particles smaller than 149 μm were selected to obtain visually homogeneous sauces. Xanthan gum was dispersed in water at 25 °C with a magnetic stirrer (Heidolph MR3001, Schwabach, Germany). Cocoa powder and the sieved sugar were then added to the aqueous phase and homogenized at 10,000 rpm for 1 min using a high-shear homogenizer (Daihan HG-15D, Gangwondo, Republic of Korea). Palm oil and sunflower oil were used as the oil phase in control formulations. Subsequently, the oil phase was incorporated into the mixture and homogenized at 10,000 rpm for 3 min to obtain chocolate sauces. The formulations of the chocolate sauce samples are presented in Table S2.

#### 2.2.3. Rheological Properties

All rheological measurements were performed using a controlled-stress rheometer (Anton Paar MCR 302, Graz, Austria) equipped with a Peltier temperature control system (Anton Paar MCR 302, Graz, Austria). A parallel plate geometry (PP50) was used with a fixed gap of 0.5 mm. All measurements were conducted at 25 °C, and each analysis was performed in triplicate.

##### Flow Behavior Rheological Properties

The flow behavior of chocolate sauce samples was evaluated over a shear rate range of 0–100 s^−1^. Approximately 2 g of sample was loaded onto the rheometer plate, and measurements were initiated after a 1 min equilibration period to ensure thermal stability. Shear stress and apparent viscosity values were recorded as a function of shear rate. Flow behavior parameters were determined by fitting the experimental data to the Power Law model using nonlinear regression (Equation (1)).

(1) $${\tau =\;K\;(\gamma )}^{n}\;$$

In the equation, τ represents the shear stress (Pa), K represents the consistency coefficient (Pas^n^), γ represents the shear rate (s^−^^1^) and n represents the flow behavior index (dimensionless).

##### Dynamic Rheological Properties

Dynamic rheological properties were evaluated by frequency sweep measurements. An amplitude sweep test (0.1–100% strain) was initially performed to determine the linear viscoelastic region (LVR). Frequency sweep tests were subsequently conducted within the LVR over a frequency range of 0.1–10 Hz (angular frequency, ω = 0.1–64 s^−1^). Storage modulus (G′) and loss modulus (G″) were recorded as functions of frequency. Dynamic rheological parameters were calculated using Equations (2) and (3) [16].

(2) $${G^{\prime }=\;K^{\prime }\;(\omega )}^{n\prime }$$

(3) $${G^{\prime \prime }=\;K^{\prime \prime }\;(\omega )}^{n\prime \prime }$$

In the equation, G′ represents the storage modulus (Pa), G″ represents the loss modulus (Pa), *ω* represents the angular velocity (s^−1^), K′ and K″ represent the coefficient of consistency (Pas^n^) and n′ and n″ represent the flow behavior index (dimensionless).

##### Temperature-Dependent Viscoelastic Properties

Temperature-dependent viscoelastic properties of emulsion and sauce samples were evaluated by temperature sweep tests. For emulsion samples, changes in the storage modulus (G′) were monitored over a temperature range of 20–100 °C, while sauce samples were analyzed between 5 and 90 °C. The temperature was increased at a constant rate of 5 °C·min^−1^.

##### Thermal Loop Test

The thermal loop test was used to evaluate the emulsion stability of oil-in-water emulsions. Although this test can be applied over both low (5–23 °C) and high (23–45 °C) temperature ranges [17], only the high-temperature range (23–45 °C) was used for the sauce samples in this study. Measurements were conducted at a fixed angular frequency of 10 Hz and a strain of 0.5%. Heating and cooling were performed at a constant rate of 11 °C·min^−1^.

#### 2.2.4. Texture Profile Analysis (TPA)

The hardness and spreadability properties of chocolate sauce samples were determined using McGill and Hartel’s [18] method with minor modifications and a texture analyser (TA.XT2 Plus, Godalming, UK). During the analysis, a 5 kg load cell and female (negative) and male (positive) acrylic 90° conical apparatus (TTC Spreadability probe-HDP/SR) were used. The chocolate sauce samples were filled into the female cone with a spatula so that no air bubbles remained, and the surface was smoothed. The reading was obtained by immersing the male cone at a speed of 3 mm/s to a depth of 23 mm from the sample surface. The rotation speed was set to 10 mm/s and the height to 25 mm. The analysis was performed at room temperature (22 °C) and repeated three times.

#### 2.2.5. Optical Microscopy

Emulsion microstructures were examined using an optical microscope equipped with a digital camera (Olympus BX41, Tokyo, Japan). A small amount of each emulsion sample was taken and placed on a slide, which was then covered with a coverslip. Micrographs were captured at 10× magnification and recorded for further analysis.

#### 2.2.6. Oxidative Stability of Chocolate Sauces

The oxidative stability of chocolate sauce samples was determined using the OXITEST instrument (Velp Scientifica, Usmate, MB, Italy). Approximately 20 g of each sample was placed into the test chambers, and analyses were performed at an oxygen pressure of 6 bar and a temperature of 90 °C. Oxidative stability was expressed as the induction period (IP), as determined by the OXITEST device [19].

#### 2.2.7. Sensory Properties of Chocolate Sauces

The sensory evaluation of chocolate sauce samples was conducted conformable to the method applied by Kasapoglu et al. [20]. During the sensory analysis, panelists were given sauce samples, unsalted crackers, and room-temperature water to rinse their mouths. The samples were assessed for odor, color, aroma, brightness, oiliness, consistency, flavor, and overall acceptability using a 9-point hedonic scale, where scores of 1–3 indicated poor, 4–6 average, 7–8 good, and 9 very good.

#### 2.2.8. Statistical Analysis

All experiments were implemented in triplicate, and the obtained results were expressed as mean ± standard deviation. Statistical analyses were performed using Minitab 21 software program with one-way analysis of variance (ANOVA, Tukey’s Test). Statistical significance was set at *p* < 0.05. The parameters of the Power Law model obtained from rheological analyses were estimated using nonlinear regression performed with Statistica software 13.6 ((Stat Soft Inc. in Tulsa, UK)).

## 3. Results and Discussion

### 3.1. Rheological Properties of Emulsions

**Figure 1:**
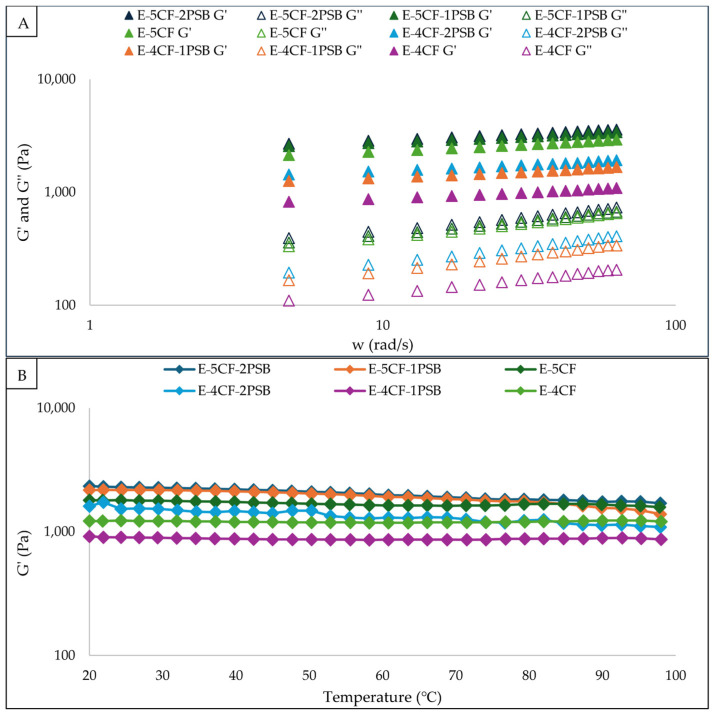
Dynamic rheological properties (**A**) and Temperature sweep test (**B**) of emulsions. E-5CF-2PSB: emulsion composed of 5% citrus fiber (CF) and 2% pumpkin seed byproduct (PSB); E-5CF-1PSB: emulsion composed of 5% CF and 1% PSB; E-5CF: emulsion composed of 5% CF; E-4CF-2PSB: emulsion composed of 4% CF and 2% PSB; E-4CF-1PSB: emulsion composed of 4% CF and 1% PSB; E-4CF: emulsion composed of 4% CF. CF: citrus fiber; PSB: pumpkin seed byproduct. prepared with palm oil; C2: control sample prepared with sunflower oil.

The dynamic rheological behavior of the emulsion samples is shown in Figure 1A. The G′ (storage modulus) values of all emulsion samples are greater than their G″ (loss modulus) values. The fact that the G′ value of each emulsion is greater than the G″ value demonstrates that the elastic character is dominant. Additionally, the G′ and G″ values of the samples show a low frequency dependence on increasing angular velocity. This is another indication of a strong gel-like structure. It was observed that the G′ value increased in parallel with the increase in CF and PSB content in the emulsion samples, and the emulsion containing 5% CF and 2% PSB had the highest G′ value. This result shows that the CF content and the presence of PSB have a remarkable effect on the viscoelastic properties of the emulsions. Similarly, in a study conducted by Genc et al. [21], it was reported that G′ and G″ values increased with increasing CF content in CF-WPI emulsions prepared using 1% to 5% CF. In a study performed by Zou et al. [22], it was determined that emulsions prepared using zein-tannic acid complex particles at different concentrations (1–5%) and sunflower oil exhibited elastic behavior because their G′ value was higher than their G″ value. Additionally, this study reported that an increase in zein-tannic acid complex particles caused an increase in the G′ value, resulting in a stronger gel structure.

The dynamic rheological parameters (K′, K″, n′, and n″ values) were modeled using the Power-Law model and are shown in Table 1. The R^2^ values of the model were found to be greater than 0.97. The K′ and K″ values of the emulsion samples vary between 694.121–2242.544 Pas^n^ and 80.693–286.086 Pas^n^. The K′ and K″ values showed a considerable increase in parallel with the increase in CF and PSB amounts. The K′ value of all emulsion samples is greater than the K″ value, demonstrating dominant solid-like viscoelastic behavior [23].

The temperature sweep test of the emulsions was conducted in the range of 20–100 °C, and the temperature-dependent G′ graphs are shown in Figure 1B. The G′ values of the emulsion samples remained relatively constant with increasing temperature, confirming the thermal stability of the solid-like viscoelastic structure in CF–PSB-containing emulsions. Similarly, in a study conducted by Bonacucina et al. [24], emulsion gels were obtained using Sepineo P 600 and almond oil at different concentrations (0.5–5%). The temperature sweep test of the emulsion gels was conducted in the range of 10–60 °C, and it was reported that there was no crucial change in the G′ value with increasing temperature. The thermal resistance of the CF–PSB emulsion network can be attributed to the complex structural composition of citrus fiber. Citrus fiber consists mainly of cellulose, hemicellulose, pectin, and protein-associated fractions, which may form a physically entangled and highly hydrated three-dimensional network. The insoluble fiber components provide a rigid structural backbone, while pectin-rich fractions contribute to water binding and intermolecular interactions. Therefore, the CF-based network can better retain its solid-like viscoelastic structure during heating, limiting thermal degradation and preventing a pronounced decrease in G′ values.

### 3.2. Rheological Properties of Chocolate Sauce

**Figure 2:**
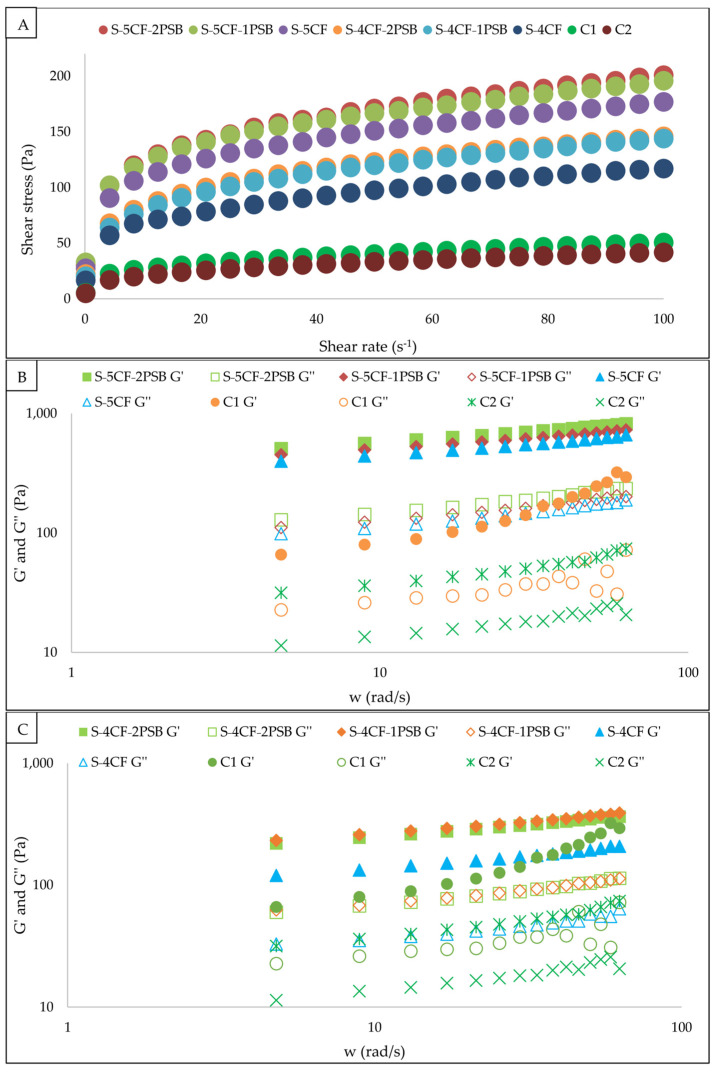

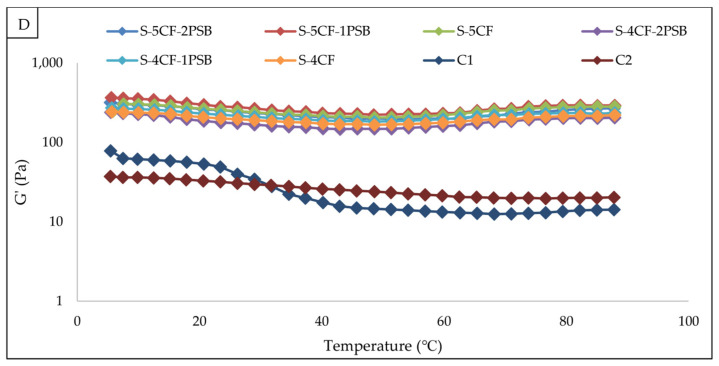
Flow behaviour rheological properties (**A**), Dynamic rheological properties (**B**,**C**), and Temperature sweep test (**D**) of chocolate sauces. S-5CF-2PSB: chocolate sauce produced using oleogel containing 5% citrus fiber (CF) and 2% pumpkin seed byproduct (PSB); S-5CF-1PSB: chocolate sauce produced using oleogel containing 5% CF and 1% PSB; S-5CF: chocolate sauce produced using oleogel containing 5% CF; S-4CF-2PSB: chocolate sauce produced using oleogel containing 4% CF and 2% PSB; S-4CF-1PSB: chocolate sauce produced using oleogel containing 4% CF and 1% PSB; S-4CF: chocolate sauce produced using oleogel containing 4% CF. C1: control sample prepared with palm oil; C2: control sample prepared with sunflower oil. CF: citrus fiber; PSB: pumpkin seed byproduct.

The flow behavior characteristics of the chocolate sauce samples are depicted in Figure 2A. The flow curves shows that all chocolate sauce samples exhibited pseudoplastic flow behavior. A diminishing increase in shear stress was detected with increasing shear rate. This finding shows a decrease in the viscosity values of the chocolate sauce samples with increasing shear rate. The decrease in viscosity can be explained by the weakening of intermolecular bonds and interactions between components in the samples under applied shear stress [25]. Similar flow behavior has been reported in previous studies for products such as fruit sauce and salad dressing [26,27]. An increase in viscosity was observed in oleogel-based sauce samples as the presence and amount of CF and PSB in the oleogel structure increased.

The parameters of flow behavior K (viscosity coefficient) and n (flow behavior index) were estimated using the Power-Law model and are shown in Table 2. The R^2^ value being greater than 0.97 demonstrates that the flow behavior rheological properties of the chocolate sauce samples can be successfully modelled using the Power-Law model. The K values of the chocolate sauce samples range significantly between 10.16 and 72.30 Pas^n^ and the n values vary between 0.22 and 0.30. An increase in the amount of CF and PSB in the oleogels contained in the chocolate sauce formulation caused an increase in the K value. An n value close to 1 signifies Newtonian flow behavior. The n values of the chocolate sauce samples being close to 0 indicate that pseudoplastic flow behavior is dominant [27]. In addition to indicating pseudoplastic flow behavior, the low n values also reflect the formation of a dense and well-structured oleogel-based matrix. Lower n values suggest greater structural organization and stronger resistance of the sauce matrix to deformation under shear. This behavior can be associated with enhanced interactions among citrus fiber, pumpkin seed oil by-product components, oil droplets, and other formulation constituents. Therefore, the simultaneous increase in K values and decrease in n values indicates that the incorporation of CF–PSB oleogels promoted a more compact and structurally reinforced matrix in the chocolate sauces. In the study by Tekin & Karasu [28], cold-pressed flaxseed oil by-product was used as a fat substitute in low-fat salad dressings. It was reported that these salad dressings exhibited pseudoplastic flow characteristics with n values in the range of 0.19–0.30, like our study, and that the K value increased as the n value decreased.

Frequency sweep tests were performed on chocolate sauce samples, and their dynamic rheological properties are demonstrated in Figure 2B,C. The G′ values of all chocolate sauce samples are greater than their G″ values. The fact that the G′ value of each chocolate sauce sample is greater than its G″ value shows that its viscoelastic solid character is dominant. Since chocolate sauces are viscoelastic solid products, the fact that the G′ value is higher than the G″ value confirms that the products possess the expected properties [25]. All oleogel-based chocolate sauces have higher G′ and G″ values compared to control (C1 and C2) samples. Additionally, oleogel-based chocolate sauce samples containing 5% CF were observed to have greater G′ and G″ values than oleogel-based chocolate sauce samples containing 4% CF. This finding is consistent with the dynamic rheological properties of CF-PSB emulsions. CF emulsions have a significant effect on the viscoelastic properties as well as the gel strength. Similarly, Chen et al. [29] reported that G′ and G″ values increase with increasing CF amount in the oleogel structure and that CF enhances gel strength.

The dynamic rheological property parameters (K′, K″, n′, and n″) of the chocolate sauce samples were estimated using the Power-Law model (Table 2). As can be presented in the table, the high R^2^ values (R^2^ > 0.97) showed that the model successfully explains the dynamic rheological properties of the samples.

The K′ and K″ values of the chocolate sauce samples range from 16.95 to 356.56 Pas^n^ and 8.04 to 87.31 Pas^n^, respectively. An increase in the amounts of CF and PSB in the oleogels present in the chocolate sauce formulation has caused a notable increase in the K′ and K″ values. The K′ value of all chocolate sauce samples is greater than the K″ value. These results show that chocolate sauce samples exhibit gel-like solid behavior. The fact that the n′ and n″ values are close to zero is another indicator that the samples have a stronger solid character. In addition to the increase in CF content of oleogels in chocolate sauce formulations, the presence of PSB was also found to enhance the gel-like solid properties. The presence of PSB in the oleogel structure caused a remarkable difference in the K′ value of chocolate sauces (*p* < 0.05). Tekin-Cakmak et al. [30] carried out a study on the use of PSB in low-fat salad dressing formulations. The researchers reported that the G′ of the dressings was greater than the G″, pointing to a dominant solid-like character. Like our work, an increase in PSB concentration resulted in higher G′ values, which strengthened the desired solid-like structure. The authors attributed this effect to the dietary fiber content of PSB.

The temperature sweep test of chocolate sauce samples was carried out in the range of 5–90 °C. Temperature-dependent G′ graphs were illustrated in Figure 2D. As shown in the figure, it has been demonstrated that the G′ value in sauce samples containing oleogel did not show dramatic change with increasing temperature. This result demonstrates that samples containing oleogel possess high thermal stability and that elastic behavior is maintained. In contrast, a dramatic decrease in the G′ value was observed with increasing temperature in C1 and C2 samples. This decrease explains that these samples exhibit a more temperature-dependent viscoelastic behavior and that the elastic character has weakened. Increasing temperature melts the C1 sauce, leading to a decrease in its storage modulus G′ and a loss of elastic behavior. This observation aligns with Naeli et al.’s [31] findings, which also reported a decrease in the G′ of interesterified palm stearin and soybean oil mixtures with rising temperatures, attributing the effect to the melting of crystallized oils.

**Figure 3:**
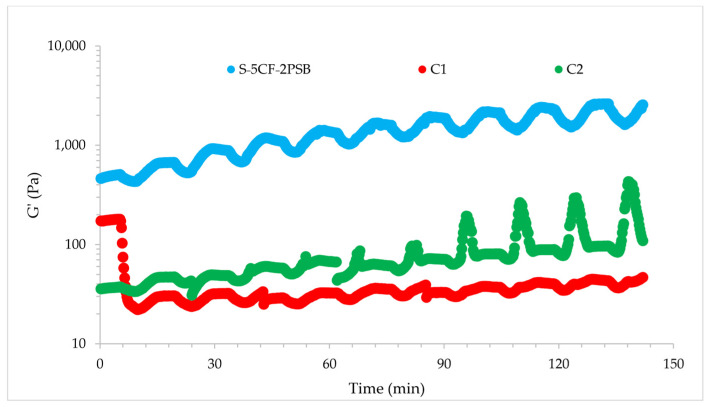
Thermal loop test of chocolate sauce. S-5CF-2PSB: chocolate sauce produced using oleogel containing 5% citrus fiber (CF) and 2% pumpkin seed byproduct (PSB). C1: control sample prepared with palm oil; C2: control sample prepared with sunflower oil. CF: citrus fiber; PSB: pumpkin seed byproduct.

Emulsion stability is an important quality criterion for sauce-like products. Chocolate sauce samples were subjected to a high-temperature (23–45 °C) thermal loop test to identify emulsion stability. The change in G′ value due to temperature change is shown in Figure 3. The high change in G′ value as a result of the applied thermal loop indicates that emulsion stability is affected by thermal loops [17]. A notable change was detected in the G′ value of the C2 control sample. This shows that the C2 sample has low stability against the applied thermal loops. There is a minimal change in the G′ value of the oleogel-containing chocolate sauce sample. This finding indicates that the use of oleogel in chocolate sauces improves emulsion stability.

### 3.3. Texture Profile Analysis (TPA)

In this study, which examined the textural properties of chocolate sauce samples, it was determined that the use of oleogel in the formulation had a noteworthy effect on the hardness and spreadability properties of the product. Both hardness and spreadability parameters increased depending on the CF and PSB content in the oleogel structure (Table 3). The textural properties of emulsions are closely associated with their rheological performance; as viscoelasticity increases, mechanical properties also improve [32]. The compatibility of the flow and viscoelastic properties with the textural properties of the chocolate sauce samples in our study is consistent with the information in the literature. Similarly to our study, a study by Szafrańska and Sołowiej [32] reported that the hardness and stickiness values of cheese sauce prepared using citrus fiber (CF) increased in parallel with the increase in CF content.

### 3.4. Optical Microscopy

**Figure 4:**
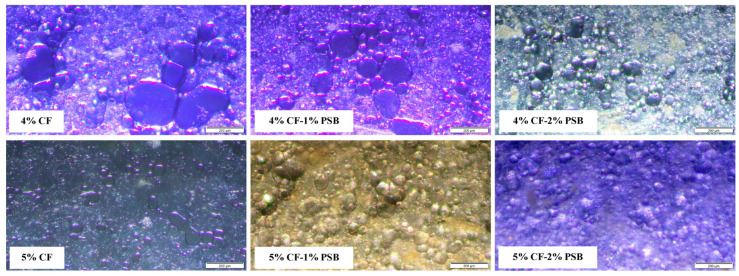
Optical microscope images of emulsions formulated with different concentrations of citrus fiber (CF) and pumpkin seed byproduct (PSB). 4% CF: emulsion composed of 4% citrus fiber; 4% CF-1% PSB: emulsion composed of 4% CF and 1% PSB; 4% CF-2% PSB: emulsion composed of 4% CF and 2% PSB; 5% CF: emulsion composed of 5% CF; 5% CF-1% PSB: emulsion composed of 5% CF and 1% PSB; 5% CF-2% PSB: emulsion composed of 5% CF and 2% PSB. CF: citrus fiber; PSB: pumpkin seed byproduct.

Optical microscope images of the emulsions are depicted in Figure 4. Oil droplet size decreased with increasing CF and PSB concentrations, indicating the formation of a more compact network structure. Similar results have been reported by Huang et al. [33] and Cui et al. [34]. These results can be attributed to the CF molecules adsorbed on the droplet interface reducing the droplet size. The decrease in droplet size leads to an increase in the viscosity and storage modulus (G′) of the emulsions [35]. In our study, the decrease in droplet size and increase in storage modulus of emulsions associated with an increase in CF and PSB ratios is consistent with the information found in the literature.

### 3.5. Oxidative Stability of Chocolate Sauces

**Figure 5:**
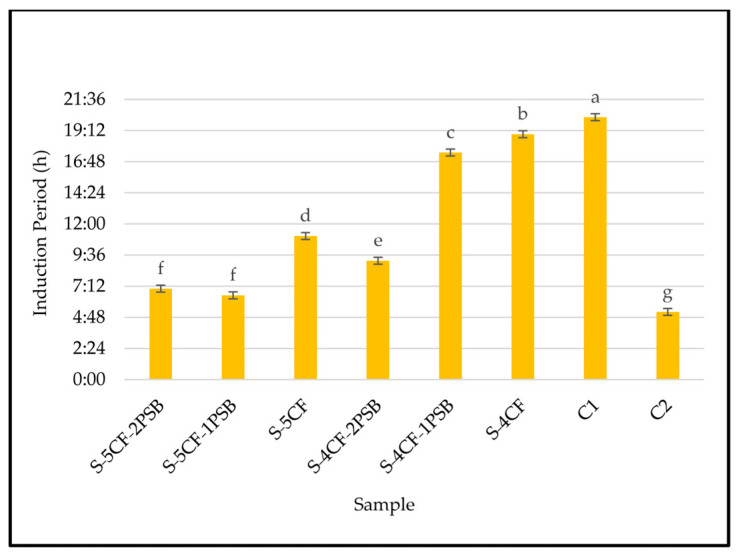
Oxidative stability of chocolate sauces. S-5CF-2PSB: chocolate sauce produced using oleogel containing 5% citrus fiber (CF) and 2% pumpkin seed byproduct (PSB); S-5CF-1PSB: chocolate sauce produced using oleogel containing 5% CF and 1% PSB; S-5CF: chocolate sauce produced using oleogel containing 5% CF; S-4CF-2PSB: chocolate sauce produced using oleogel containing 4% CF and 2% PSB; S-4CF-1PSB: chocolate sauce produced using oleogel containing 4% CF and 1% PSB; S-4CF: chocolate sauce produced using oleogel containing 4% CF. C1: control sample prepared with palm oil; C2: control sample prepared with sunflower oil. CF: citrus fiber; PSB: pumpkin seed byproduct. ^a–g^: Different superscript letters indicate significant differences between samples (*p* < 0.05).

Induction period (IP) values were used to evaluate the oxidative stability of chocolate sauces (Figure 5). As expected, the palm oil control (C1) exhibited the highest oxidative stability (20:13 h), whereas the sunflower oil control (C2) showed the lowest IP value (5:13 h), confirming the superior oxidative resistance of palm oil. All oleogel-based chocolate sauces displayed significantly higher oxidative stability than the sunflower oil control, demonstrating the effectiveness of the emulsion-templated oleogel structure in protecting the lipid phase against oxidation. Among the oleogel-containing formulations, the S-4CF sample exhibited the highest IP value (18:55 h), representing the closest oxidative stability to the palm oil control. Although none of the oleogel-based formulations achieved oxidative stability statistically equivalent to C1, the relatively small difference between S-4CF and C1 suggests that CF-based oleogels can partially mimic the oxidative protection provided by palm oil. These findings indicate the potential of emulsion-templated oleogels as sustainable alternatives to conventional solid fats in chocolate sauce formulations. In contrast, formulations containing 5% CF generally exhibited lower IP values than the corresponding 4% CF samples, suggesting that increasing CF concentration beyond a certain level may promote pro-oxidative effects. Similar observations were reported by Gedikoglu et al. [36], who found that the incorporation of CF at 3% and 5% levels promoted oxidation in beef patties. Likewise, Hamidioglu et al. [37] reported that increasing rice bran wax concentration in rice bran wax–candelilla wax oleogels adversely affected the oxidative stability of hemp oil, attributing this behavior to the pro-oxidative effect of the wax network. Furthermore, the incorporation of pumpkin seed oil by-product (PSB) into the oleogel structure resulted in lower IP values compared with the corresponding formulations without PSB. This effect may be attributed to the high content of unsaturated fatty acids associated with pumpkin seed components. Jakab et al. [38] reported that pumpkin seed oil is particularly rich in unsaturated fatty acids, especially linoleic acid, which may increase susceptibility to lipid oxidation. Therefore, although PSB contributes nutritional and sustainability benefits, its incorporation may adversely affect the oxidative stability of oleogel-based systems.

### 3.6. Sensory Properties of Chocolate Sauces

**Figure 6:**
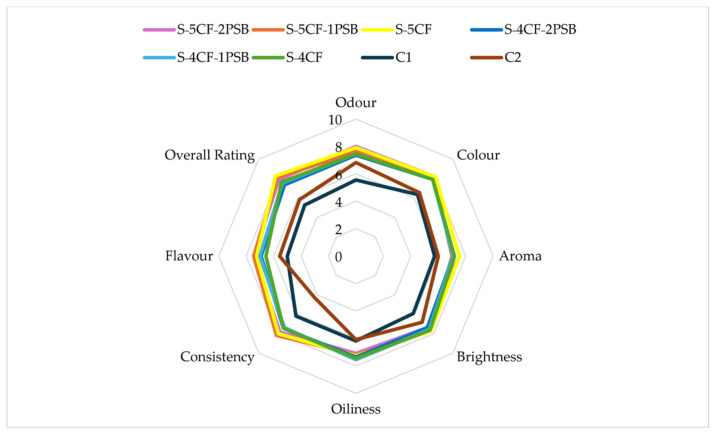
Sensory properties of chocolate sauces. S-5CF-2PSB: chocolate sauce produced using oleogel containing 5% citrus fiber (CF) and 2% pumpkin seed byproduct (PSB); S-5CF-1PSB: chocolate sauce produced using oleogel containing 5% CF and 1% PSB; S-5CF: chocolate sauce produced using oleogel containing 5% CF; S-4CF-2PSB: chocolate sauce produced using oleogel containing 4% CF and 2% PSB; S-4CF-1PSB: chocolate sauce produced using oleogel containing 4% CF and 1% PSB; S-4CF: chocolate sauce produced using oleogel containing 4% CF. C1: control sample prepared with palm oil; C2: control sample prepared with sunflower oil.

Sensory evaluation results of chocolate sauce samples are presented in Figure 6. Overall acceptability scores ranged from 5.27 ± 1.56 to 8.27 ± 0.90. The S-5% CF sample received the highest overall rating, whereas the lowest score was observed for the C1 sample. The S-5%CF-2% PSB formulation exhibited the highest aroma acceptability (8.00 ± 0.89). No statistically significant differences were detected among samples in terms of color, aroma, brightness, and oiliness (*p* > 0.05). The product with the most preferred consistency was S-5% CF-1% PSB (8.18 ± 0.87), while the product with the least preferred consistency was the C2 (4.27 ± 1.74) sample. The highest flavor preference was observed for the S-5%CF–2%PSB and S-5%CF–1%PSB samples (both 7.45), whereas the C1 sample received the lowest flavor score (5.00 ± 2.57). Overall, oleogel-containing chocolate sauces were more favorably perceived by panelists than the control formulations.

## 4. Conclusions

Oleogels were produced using the emulsion-template method with CF and PSB and evaluated as palm oil substitutes in a model chocolate sauce system. The increase in CF and PSB concentrations enhanced the network strength and resulted in oleogels with higher viscoelastic moduli and improved textural properties. When incorporated into chocolate sauce formulations, the CF–PSB emulsion-templated oleogels provided rheological behavior, texture, and sensory characteristics comparable or superior to palm oil. In addition, CF–PSB emulsion-templated oleogel-based sauces showed improved oxidative stability relative to sunflower oil formulations and maintained emulsion stability under thermal stress. Sensory evaluation indicated high acceptability for sauces containing 5% CF and 1–2% PSB. CF–PSB emulsion-templated oleogels represent a promising plant-based and sustainable fat-structuring strategy for a model chocolate sauce system, enabling the reduction in saturated fat content without compromising product quality.

## Figures and Tables

**Table 1:** The power law parameters of dynamic rheological properties of emulsions.

Sample	K′ (Pas^n^)	n′	R^2^	K″ (Pas^n^)	n″	R^2^
E-5CF-2PSB	2242.5 ^a^ ± 41.46	0.108 ^b^ ± 0.000	>0.99	286.1 ^a^ ± 3.21	0.216 ^c^ ± 0.002	0.98
E-5CF-1PSB	2126.7 ^a^ ± 13.66	0.104 ^b^ ± 0.001	>0.99	257.5 ^ab^ ± 1.47	0.219 ^c^ ± 0.002	0.98
E-5CF	1776.5 ^b^ ± 6.73	0.118 ^a^ ± 0.002	>0.99	244.1 ^b^ ± 4.89	0.232 ^bc^ ± 0.004	0.98
E-4CF-2PSB	1241.9 ^c^ ± 11.12	0.104 ^b^ ± 0.003	>0.99	137.2 ^c^ ± 7.21	0.262 ^a^ ± 0.008	0.99
E-4CF-1PSB	1076.0 ^c^ ± 7.75	0.103 ^b^ ± 0.004	>0.99	122.0 ^cd^ ± 7.72	0.243 ^ab^ ± 0.015	0.98
E-4CF	694.1 ^d^ ± 9.45	0.101 ^b^ ± 0.003	>0.99	80.7 ^d^ ± 5.05	0.213 ^c^ ± 0.008	0.97

^a–d^: Different superscript letters indicate significant differences between samples (*p* < 0.05). E-5CF-2PSB: emulsion composed of 5% citrus fiber (CF) and 2% pumpkin seed byproduct (PSB); E-5CF-1PSB: emulsion composed of 5% CF and 1% PSB; E-5CF: emulsion composed of 5% CF; E-4CF-2PSB: emulsion composed of 4% CF and 2% PSB; E-4CF-1PSB: emulsion composed of 4% CF and 1% PSB; E-4CF: emulsion composed of 4% CF. CF: citrus fiber; PSB: pumpkin seed byproduct.

**Table 2:** Rheological parameters of the chocolate sauces.

Sample	K (Pas^n^)	n	R^2^	K′ (Pas^n^)	n′	R^2^	K″ (Pas^n^)	n″	R^2^
S-5CF-2PSB	72.30 ^a^ ± 0.41	0.22 ^e^ ± 0.00	>0.99	356.56 ^a^ ± 14.61	0.19 ^b^ ± 0.00	>0.99	87.31 ^a^ ± 6.32	0.22 ^bc^ ± 0.01	>0.97
S-5CF-1PSB	65.48 ^b^ ± 3.01	0.23 ^de^ ± 0.00	>0.99	350.44 ^ab^ ± 18.49	0.19 ^b^ ± 0.00	>0.99	84.15 ^a^ ± 8.68	0.22 ^bc^ ± 0.01	>0.97
S-5CF	65.29 ^b^ ± 1.84	0.22 ^e^ ± 0.00	>0.99	297.91 ^b^ ± 9.93	0.19 ^b^ ± 0.00	>0.99	70.78 ^a^ ± 2.97	0.23 ^bc^ ± 0.00	>0.97
S-4CF-2PSB	48.23 ^cd^ ± 1.74	0.26 ^c^ ± 0.00	>0.99	150.39 ^c^ ± 11.28	0.20 ^b^ ± 0.00	>0.99	39.02 ^bc^ ± 3.35	0.24 ^bc^ ± 0.01	>0.97
S-4CF-1PSB	43.31 ^c^ ± 0.16	0.24 ^cd^ ± 0.00	>0.99	168.27 ^c^ ± 0.97	0.20 ^b^ ± 0.00	>0.99	45.28 ^b^ ± 0.59	0.21 ^c^ ± 0.01	>0.97
S-4CF	38.18 ^d^ ± 1.13	0.24 ^cd^ ± 0.00	>0.99	89.32 ^d^ ± 8.95	0.21 ^b^ ± 0.00	>0.99	25.04 ^cd^ ± 3.64	0.21 ^c^ ± 0.02	>0.97
C1	12.98 ^e^ ± 0.62	0.28 ^b^ ± 0.00	>0.99	34.84 ^de^ ± 1.41	0.34 ^a^ ± 0.05	>0.99	15.40 ^d^ ± 4.03	0.25 ^ab^ ± 0.17	>0.97
C2	10.16 ^e^ ± 0.16	0.30 ^a^ ± 0.00	>0.99	16.95 ^e^ ± 0.34	0.36 ^a^ ± 0.03	>0.99	8.04 ^d^ ± 0.02	0.27 ^a^ ± 0.01	>0.97

^a–e^: Different superscript letters indicate significant differences between samples (*p* < 0.05). S-5CF-2PSB: chocolate sauce produced using oleogel containing 5% citrus fiber (CF) and 2% pumpkin seed byproduct (PSB); S-5CF-1PSB: chocolate sauce produced using oleogel containing 5% CF and 1% PSB; S-5CF: chocolate sauce produced using oleogel containing 5% CF; S-4CF-2PSB: chocolate sauce produced using oleogel containing 4% CF and 2% PSB; S-4CF-1PSB: chocolate sauce produced using oleogel containing 4% CF and 1% PSB; S-4CF: chocolate sauce produced using oleogel containing 4% CF. C1: control sample prepared with palm oil; C2: control sample prepared with sunflower oil. CF: citrus fiber; PSB: pumpkin seed byproduct.

**Table 3:** Textural properties of chocolate sauces.

Sample	Hardness (g)	Spreadability (g.s)
S-5CF-2PSB	264.16 ^a^ ± 2.79	246.74 ^a^ ± 2.82
S-5CF-1PSB	245.32 ^a^ ± 4.27	198.19 ^b^ ± 4.18
S-5CF	242.82 ^a^ ± 1.28	193.20 ^b^ ± 5.58
S-4CF-2PSB	187.77 ^b^ ± 4.22	160.04 ^c^ ± 2.23
S-4CF-1PSB	181.81 ^b^ ± 1.95	127.60 ^d^ ± 0.08
S-4CF	180.64 ^b^ ± 1.25	118.22 ^d^ ± 2.54
C1	70.46 ^c^ ± 1.03	58.94 ^e^ ± 0.06
C2	50.52 ^c^ ± 1.34	28.24 ^e^ ± 0.13

^a–e^: Different superscript letters indicate significant differences between samples (*p* < 0.05). S-5CF-2PSB: chocolate sauce produced using oleogel containing 5% citrus fiber (CF) and 2% pumpkin seed byproduct (PSB); S-5CF-1PSB: chocolate sauce produced using oleogel containing 5% CF and 1% PSB; S-5CF: chocolate sauce produced using oleogel containing 5% CF; S-4CF-2PSB: chocolate sauce produced using oleogel containing 4% CF and 2% PSB; S-4CF-1PSB: chocolate sauce produced using oleogel containing 4% CF and 1% PSB; S-4CF: chocolate sauce produced using oleogel containing 4% CF. C1: control sample prepared with palm oil; C2: control sample prepared with sunflower oil. CF: citrus fiber; PSB: pumpkin seed byproduct.

## Data Availability

The original contributions presented in the study are included in the article/Supplementary Material, further inquiries can be directed to the corresponding authors.

## Supplementary Materials

The following supporting information can be downloaded at: https://www.mdpi.com/article/10.3390/foods15132272/s1, Table S1: Formulation of hydrogels. H-5CF-2PSB: hydrogel composed of 5% citrus fiber (CF) and 2% pumpkin seed byproduct (PSB); H-5CF-1PSB: hydrogel composed of 5% CF and 1% PSB; H-5CF: hydrogel composed of 5% CF; H-4CF-2PSB: hydrogel composed of 4% CF and 2% PSB; H-4CF-1PSB: hydrogel composed of 4% CF and 1% PSB; H-4CF: hydrogel composed of 4% CF. Table S2. Formulation of chocolate sauces. S-5CF-2PSB: chocolate sauce produced using oleogel containing 5% citrus fiber (CF) and 2% pumpkin seed byproduct (PSB); S-5CF-1PSB: chocolate sauce produced using oleogel containing 5% CF and 1% PSB; S-5CF: chocolate sauce produced using oleogel containing 5% CF; S-4CF-2PSB: chocolate sauce produced using oleogel containing 4% CF and 2% PSB; S-4CF-1PSB: chocolate sauce produced using oleogel containing 4% CF and 1% PSB; S-4CF: chocolate sauce produced using oleogel containing 4% CF. C1: control sample prepared with palm oil; C2: control sample prepared with sunflower oil.

## Abbreviations

The following abbreviations are used in this manuscript:

CF	Citrus fiber
PSB	Pumpkin seed oil by-product
C1	Control samples prepared from palm oil
C2	Control samples prepared from sunflower oil
G′	Storage modulus
G″	Loss modulus
K′ and K″	Coefficient of consistency
LVR	Linear viscoelastic region
n′ and n″	Flow behavior index
ANOVA	Analysis of variance
*ω*	Angular velocity

## References

[1] Pinto T.C., Martins A.J., Pastrana L., Pereira M.C., Cerqueira M.A. (2021). Oleogel-Based Systems for the Delivery of Bioactive Compounds in Foods. Gels.

[2] Martins A.J., Vicente A.A., Pastrana L.M., Cerqueira M.A. (2020). Oleogels for development of health-promoting food products. Food Sci. Hum. Wellness.

[3] Islam M.A., Amin M.N., Siddiqui S.A., Hossain M.P., Sultana F., Kabir M.R. (2019). Trans fatty acids and lipid profile: A serious risk factor to cardiovascular disease, cancer and diabetes. Diabetes Metab. Syndr. Clin. Res. Rev..

[4] Nutter J., Shi X., Lamsal B., Acevedo N.C. (2023). Designing and characterizing multicomponent, plant-based bigels of rice bran wax, gums, and monoglycerides. Food Hydrocoll..

[5] Wang Q., Espert M., Flores M., Sanz T., Salvador A. (2025). Oxidative and texture storage stability of HPMC sunflower oil oleogels prepared by different indirect approaches. Food Hydrocoll..

[6] Sabet S., Pinto T.C., Kirjoranta S.J., Garcia A.K., Valoppi F. (2023). Clustering of oleogel production methods reveals pitfalls and advantages for sustainable, upscalable, and oxidative stable oleogels. J. Food Eng..

[7] Abdollahi M., Goli S.A.H., Soltanizadeh N. (2020). Physicochemical properties of foam-templated oleogel based on gelatin and xanthan gum. Eur. J. Lipid Sci. Technol..

[8] Espert M., Hernández M.J., Sanz T., Salvador A. (2022). Rheological properties of emulsion templated oleogels based on xanthan gum and different structuring agents. Curr. Res. Food Sci..

[9] Wallecan J., McCrae C., Debon S., Dong J., Mazoyer J. (2015). Emulsifying and stabilizing properties of functionalized orange pulp fibers. Food Hydrocoll..

[10] Huang J.-Y., Liao J.-S., Qi J.-R., Jiang W.-X., Yang X.-Q. (2021). Structural and physicochemical properties of pectin-rich dietary fiber prepared from citrus peel. Food Hydrocoll..

[11] He C.-A., Qi J.-R., Liao J.-S., Song Y.-T., Wu C.-L. (2023). Excellent hydration properties and oil holding capacity of citrus fiber: Effects of component variation and microstructure. Food Hydrocoll..

[12] Tekin-Cakmak Z.H., Karasu S., Kayacan-Cakmakoglu S., Akman P.K. (2021). Investigation of potential use of by-products from cold-press industry as natural fat replacers and functional ingredients in a low-fat salad dressing. J. Food Process. Preserv..

[13] Guadalupe G.A., Lerma-García M.J., Fuentes A., Barat J.M., Bas M.D.C., Fernández-Segovia I. (2019). Presence of palm oil in foodstuffs: Consumers’ perception. Br. Food J..

[14] Yılmaz B., Ağagündüz D. (2022). Fractionated palm oils: Emerging roles in the food industry and possible cardiovascular effects. Crit. Rev. Food Sci. Nutr..

[15] Qiu C., Huang Y., Li A., Ma D., Wang Y. (2018). Fabrication and Characterization of Oleogel Stabilized by Gelatin-Polyphenol-Polysaccharides Nanocomplexes. J. Agric. Food Chem..

[16] Yoo B., Rao M. (1996). Creep and dynamic rheological behavior of tomato concentrates: Effect of concentration and finisher screen size. J. Texture Stud..

[17] Tekin Z.H., Avci E., Karasu S., Toker O.S. (2020). Rapid determination of emulsion stability by rheology-based thermal loop test. LWT.

[18] Mcgill J., Hartel R.W. (2018). Investigation into the microstructure, texture and rheological properties of chocolate ganache. J. Food Sci..

[19] Avci E., Akcicek A., Tekin Cakmak Z.H., Kasapoglu M.Z., Sagdic O., Karasu S. (2024). Isolation of Protein and Fiber from Hot Pepper Seed Oil Byproduct To Enhance Rheology, Emulsion, and Oxidative Stability of Low-Fat Salad Dressing. ACS Omega.

[20] Kasapoglu M.Z., Sagdic O., Avci E., Tekin-Cakmak Z.H., Karasu S., Turker R.S. (2023). The potential use of cold-pressed coconut oil by-product as an alternative source in the production of plant-based drink and plant-based low-fat ice cream: The rheological, thermal, and sensory properties of plant-based ice cream. Foods.

[21] Genc E., Karasu S., Akcicek A., Toker O.S. (2024). Fabrication and characterisation of Pickering emulsion-based oleogel stabilised by citrus fibre and whey protein isolate colloidal complex: Application in cookie formulation. Int. J. Food Sci. Technol..

[22] Zou Y., Yang X., Scholten E. (2018). Rheological behavior of emulsion gels stabilized by zein/tannic acid complex particles. Food Hydrocoll..

[23] Atik I., Tekin Cakmak Z.H., Avcı E., Karasu S. (2021). The effect of cold press chia seed oil by-products on the rheological, microstructural, thermal, and sensory properties of low-fat ice cream. Foods.

[24] Bonacucina G., Cespi M., Palmieri G.F. (2009). Characterization and stability of emulsion gels based on acrylamide/sodium acryloyldimethyl taurate copolymer. AAPS PharmSciTech.

[25] Avci E., Tekin-Cakmak Z.H., Ozgolet M., Karasu S., Kasapoglu M.Z., Ramadan M.F., Sagdic O. (2023). Capsaicin Rich Low-Fat Salad Dressing: Improvement of Rheological and Sensory Properties and Emulsion and Oxidative Stability. Foods.

[26] Yalçınöz Ş.K., Erçelebi E. (2016). Rheological and sensory properties of red colored fruit sauces prepared with different hydrocolloids. J. Int. Sci. Publ. Agric. Food.

[27] Akcicek A., Karasu S. (2018). Utilization of cold pressed chia seed oil waste in a low-fat salad dressing as natural fat replacer. J. Food Process Eng..

[28] Tekin Z.H., Karasu S. (2020). Cold-pressed flaxseed oil by-product as a new source of fat replacers in low-fat salad dressing formulation: Steady, dynamic and 3-ITT rheological properties. J. Food Process. Preserv..

[29] Chen X.-W., Zhang H., Li X.-X., Sun S.-D. (2023). Edible HIPE-Gels and oleogels formed by synergistically combining natural triterpenoid saponin and citrus dietary fiber. Carbohydr. Polym..

[30] Tekin-Cakmak Z.H., Atik I., Karasu S. (2021). The potential use of cold-pressed pumpkin seed oil by-products in a low-fat salad dressing: The effect on rheological, microstructural, recoverable properties, and emulsion and oxidative stability. Foods.

[31] Naeli M.H., Farmani J., Zargaraan A. (2017). Rheological and Physicochemical Modification of trans-Free Blends of Palm Stearin and Soybean Oil by Chemical Interesterification. J. Food Process Eng..

[32] Szafrańska J.O., Sołowiej B.G. (2020). Effect of different fibres on texture, rheological and sensory properties of acid casein processed cheese sauces. Int. J. Food Sci. Technol..

[33] Huang L., Cai Y., Fang D., Su J., Zhao M., Zhao Q., Van Der Meeren P. (2023). Formation and characterization of oleogels derived from emulsions: Evaluation of polysaccharide ratio and emulsification method. Food Hydrocoll..

[34] Cui F., Zhao S., Guan X., Mcclements D.J., Liu X., Liu F., Ngai T. (2021). Polysaccharide-based Pickering emulsions: Formation, stabilization and applications. Food Hydrocoll..

[35] Pal R. (1996). Effect of droplet size on the rheology of emulsions. AIChE J..

[36] Gedikoğlu A., Clarke A.D., Lin M., Yılmaz B. (2021). Antioxidant properties of citrus fibre and the prediction of oxidation in ground beef meatballs made with citrus fibre by ATR-FTIR spectroscopy with principal component analysis. Int. Food Res. J..

[37] Hamidioglu I., Alenčikienė G., Dzedulionytė M., Zabulionė A., Bali A., Šalaševičienė A. (2022). Characterization of the Quality and Oxidative Stability of Hemp-Oil-Based Oleogels as an Animal Fat Substitute for Meat Patties. Foods.

[38] Jakab I., Mardani M., Tormási J., Abrankó L., Badak-Kerti K. (2025). Physicochemical Characteristics of Cold-Pressed Hemp, Flax, Hazelnut, and Pumpkin Seed Oils and Press Cakes. Eur. J. Lipid Sci. Technol..

