# Supplementary material for: Emulsion-Templated Oleogels from Citrus Fiber and Pumpkin Seed Oil By-Product as Palm Oil Substitutes in Chocolate Sauce
Source: Foods. 2026 Jun 25;15(13):2272. doi: 10.3390/foods15132272 (PMC13361680; doi:10.3390/foods15132272)
Supplement: Supplementary file 1 [file foods-15-02272-s001.zip › foods-4355467-supplementary.pdf]

Table S1. Formulation of hydrogels

Sample	CF (%)	PSB (%)
H-5CF-2PSB	5	2
H-5CF-1PSB	5	1
H-5CF	5	0
H-4CF-2PSB	4	2
H-4CF-1PSB	4	1
H-4CF	4	0

H-5CF-2PSB: hydrogel composed of 5% citrus fiber (CF) and 2% pumpkin seed byproduct (PSB); H-5CF-1PSB: hydrogel composed of 5% CF and 1% PSB; H-5CF: hydrogel composed of 5% CF; H-4CF-2PSB: hydrogel composed of 4% CF and 2% PSB; H-4CF-1PSB: hydrogel composed of 4% CF and 1% PSB; H-4CF: hydrogel composed of 4% CF. CF: citrus fiber; PSB: pumpkin seed byproduct.

Table S2. Formulation of chocolate sauces

**Sample**	**Ground Powdered Sugar (%)**	**Cocoa (%)**	**Xanthan Gum (%)**	**Oleogel (%)**	**Palm Oil (%)**	**Sunflower Oil (%)**
**S-5CF-2PSB**	20	10	0.30	20	-	-
**S-5CF-1 PSB**	20	10	0.30	20	-	-
**S-5CF**	20	10	0.30	20	-	-
**S-4CF-2 PSB**	20	10	0.30	20	-	-
**S-4CF-1PSB**	20	10	0.30	20	-	-
**S-4CF**	20	10	0.30	20	-	-
**C1**	20	10	0.30	-	20	-
**C2**	20	10	0.30	-	-	20

S-5CF-2PSB: chocolate sauce produced using oleogel containing 5% citrus fiber (CF) and 2% pumpkin seed byproduct (PSB); S-5CF-1PSB: chocolate sauce produced using oleogel containing 5% CF and 1% PSB; S-5CF: chocolate sauce produced using oleogel containing 5% CF; S-4CF-2PSB: chocolate sauce produced using oleogel containing 4% CF and 2% PSB; S-4CF-1PSB: chocolate sauce produced using oleogel containing 4% CF and 1% PSB; S-4CF: chocolate sauce produced using oleogel containing 4% CF. C1: control sample prepared with palm oil; C2: control sample prepared with sunflower oil. CF: citrus fiber; PSB: pumpkin seed byproduct.
